# The CRL3^BTBD9^ E3 ubiquitin ligase complex targets TNFAIP1 for degradation to suppress cancer cell migration

**DOI:** 10.1038/s41392-020-0140-z

**Published:** 2020-04-24

**Authors:** Lihui Li, Wenjuan Zhang, Yue Liu, Xiaojun Liu, Lili Cai, Jihui Kang, Yunjing Zhang, Wenlian Chen, Changsheng Dong, Yanmei Zhang, Mingsong Wang, Wenyi Wei, Lijun Jia

**Affiliations:** 1 Cancer Institute, Longhua Hospital, Shanghai University of Traditional Chinese Medicine, Shanghai, China; 2 Cancer Institute, Fudan University Shanghai Cancer Center, Fudan University, Shanghai, China; 3 Department of Laboratory Medicine, Huadong Hospital, Affiliated to Fudan University, Shanghai, China; 4 Xinhua Hospital, Shanghai Jiaotong University, Shanghai, China; 5 Department of Pathology, Beth Israel Deaconess Medical Center, Harvard Medical School, Boston, MA USA

**Keywords:** Lung cancer, Oncogenes

## Abstract

Tumor necrosis factor alpha-induced protein 1 (TNFAIP1) modulates a plethora of important biological processes, including tumorigenesis and cancer cell migration. However, the regulatory mechanism of TNFAIP1 degradation remains largely elusive. In the present study, with a label-free quantitative proteomic approach, TNFAIP1 was identified as a novel ubiquitin target of the Cullin-RING E3 ubiquitin ligase (CRL) complex. More importantly, Cul3-ROC1 (CRL3), a subfamily of CRLs, was identified to specifically interact with TNFAIP1 and promote its polyubiquitination and degradation. Mechanistically, BTBD9, a specific adaptor component of CRL3 complex, was further defined to bind and promote the ubiquitination and degradation of TNFAIP1 in cells. As such, downregulation of BTBD9 promoted lung cancer cell migration by upregulating the expression of TNFAIP1, whereas TNFAIP1 deletion abrogated this effect. Finally, bioinformatics and clinical sample analyses revealed that BTBD9 was downregulated while TNFAIP1 was overexpressed in human lung cancer, which was associated with poor overall survival of patients. Taken together, these findings reveal a previously unrecognized mechanism by which the CRL3^BTBD9^ ubiquitin ligase controls TNFAIP1 degradation to regulate cancer cell migration.

## Introduction

Cullin-RING ligases (CRLs) are multi-subunit E3 ubiquitin ligase complexes that regulate the ubiquitination and subsequent degradation of ~20% of proteins degraded by the ubiquitin-proteasome system, including transcription factors, tumor suppressors, and oncoproteins.^1,2^ Based on different Cullin proteins (Cul 1–3, Cul 4A/4B, Cul 5, Cul 7, and Cul 9), CRL complexes are divided into eight subfamilies, and most of them are consisted of Cullin proteins, adaptor proteins, substrate receptor proteins, and Ring-finger proteins.^3,4^ Among them, Cul3-RING ligases (CRL3s) are well studied E3 ubiquitin ligases consisting of Cul3 as the scaffold in complex with Ring-finger protein RING-box protein 1 (ROC1) at the C-terminus of Cul3 and substrate specific Bric-a-Brac/Tramtrack/Broad (BTB) domain-containing proteins at the N-terminus.^5–7^ CRLs are dysregulated in many types of cancers, thus leading to the accelerated degradation of tumor suppressors and promoting tumorigenesis and tumor progression.^8–12^

The activation of CRLs requires the covalent binding of NEDD8 to scaffold Cullin proteins, which is defined as a post-translational neddylation modification.^13,14^ Specifically, protein neddylation is a 3-step enzymatic cascade reaction involving NEDD8 activating enzyme E1 (NAE, a heterodimer consisting of NAE1 and UBA3), NEDD8-conjugating enzyme E2 (2 members, UBE2M/UBC12 and UBE2F), and NEDD8 E3 ligases.^9,15,16^ NEDD8 modification regulates a variety of biological processes via affecting the subcellular localization, stability, conformation and function of neddylated substrates.^17,18^ As a small-molecule inhibitor of NAE, MLN4924 blocks CRL activity, subsequently leading to the accumulation of an abundance of CRL E3 substrates and retarding tumor cell growth in vitro and in vivo.^1,15,19–22^

Tumor necrosis factor, alpha-induced protein 1 (TNFAIP1) was originally identified to be induced by tumor necrosis factor alpha (TNFα) in umbilical vein endothelial cells.^23^ As an extremely evolutionarily conserved single-copy gene, TNFAIP1 has important physiological roles. Functioning as a substrate specific adaptor, TNFAIP1 is involved in regulation of cytoskeleton structure by mediating the polyubiquitination and degradation of RhoA.^24^ In addition, TNFAIP1 is involved in DNA synthesis and DNA repair in part through directly interacting with proliferating cell nuclear antigen (PCNA) and the small subunit (p50) of DNA polymerase δ.^25^ TNFAIP1 is also involved in multiple pathological processes, including tumorigenesis.^26,27^ Moreover, TNFAIP1 competes with paclitaxel for β-tubulin binding, thereby preventing paclitaxel-induced tubulin polymerization, cell cycle arrest and ultimately cancer cell death.^28^ Although TNFAIP1 plays an important role in many pivotal biological processes, the molecular mechanism underlying its degradation has not yet been fully elucidated.

In the present study, we determined the mechanism of TNFAIP1 degradation and identified the Cul3-ROC1-BTBD9 complex as a novel upstream E3 ligase targeting TNFAIP1 for polyubiquitination and subsequent degradation. Moreover, BTBD9 was shown to suppress cancer cell migration by triggering TNFAIP1 degradation. Finally, as the specific adaptor of TNFAIP1, BTBD9 was found to be expressed at low levels in lung cancer, leading to the dysregulation of CRL3^BTBD9^ and subsequent upregulation of TNFAIP1. Taken together, our findings reveal a previously unknown mechanism by which TNFAIP1 is upregulated in cancer and suggest TNFAIP1 as a potential target of cancer cell metastasis.

## Materials and methods

### Cell lines and reagents

The human cancer cell lines Huh7, HepG2, A549, and H1299 were obtained from the American Type Culture Collection and cultured in DMEM or RPMI-1640 medium supplemented with 10% FBS and 1% penicillin-streptomycin at 37 °C with 5% CO_2_. MLN4924 was synthesized and used as previously described. MG132 (Sigma Aldrich, St Louis, MO, USA) and cycloheximide (CHX, Cell Signaling Technology, Beverly, MA, USA) were kept at −20 °C.

### Protein extraction, digestion, and label-free quantification in HepG2 cells

HepG2 extracts were prepared according to our published methods.^29^ Label-free quantification was carried out by the Beijing Proteome Research Center (Beijing, China).

### RNA interference

To knockdown the ROC and Cullin family members, cells were transfected with siRNA oligonucleotides (GenePharma, Shanghai, China) using Lipofectamine 2000 reagent (Life Technologies, Invitrogen, CA, USA) according to the manufacturer’s instructions. Details are provided in the Supplementary Materials and Methods.

### Western blot and CHX chase analyses

Cell lysates were prepared for western blot analysis using antibodies against TNFAIP1 (Arigo, Cambridge, MA); ROC1, Cul1, Cul2, Cul5, and Cul7 (Abcam, Cambridge, MA, USA); Cul4b (Proteintech, Chicago, IL, USA); ubiquitin, Cul3 and Cul4a (Cell Signaling Technology, Beverly, MA, USA); β-actin as the loading control (Kangwei, Shanghai, China). To determine the half-life of TNFAIP1, cells were treated with 50 μg/mL CHX (Sigma Aldrich, St Louis, MO, USA) for the indicated durations.

### Generation of stable cell lines by the CRISPR/Cas9 system

The CRISPR/Cas9 system was used to knockdown TNFAIP1 and BTBD9. Lentivirus packaging in HEK-293T cells and the infection of A549 and H1299 cells were carried out with our published methods.^30^ More details are provided in the Supplementary Materials and Methods.

### In vivo ubiquitination assays

TNFAIP1 ubiquitination analysis, including MLN4924 treatment, transfection with siRNA against *ROC1* or *Cul3*, and transfection with sgRNA against *BTBD9*, was performed in liver and lung cancer cells following our published methods.^29^

### Transwell migration assays

For cell migration assays, TNFAIP1- or BTBD9-knockdown cells were added to the upper chambers. DMEM containing 20% FBS was added to the lower chambers. After incubation for 24 h, the upper chambers were stained with crystal violet, and cells that had passed through the membrane were counted under a Leica microscope.^30^ The representative results of three independent experiments with similar trends are presented.

### Histologic evaluation

Human lung cancer tissue arrays were subjected to immunohistochemical (IHC) staining with anti-TNFAIP1 antibody from Shanghai Biochip (Shanghai, China). IHC staining was performed with our published methods.^31^ The stained slides were observed under a microscope, and images were acquired and quantitatively classified based on staining intensity as described previously.^31–33^ Based on the staining intensity, we classified the samples into five groups: the weakest group (±), weak group (+), medium group (++), strong group (+++), and strongest group (++++). We defined the ±, +, and ++ groups as low-expression groups and the +++ and ++++ groups as high-expression groups. The collection of lung cancer tissues and clinicopathological characteristics of the patients are shown in the supplementary materials and methods. This study was approved by the Research Ethics Committee of Taizhou Hospital.

### Statistical analysis

Survival was analyzed using the Kaplan–Meier method, and data were compared using the log-rank test with Statistical Program for Social Sciences (SPSS, IBM, USA) software version 16.0. The overall survival time was defined as the duration from the date of diagnosis to the date of either death or censoring (which could occur by either loss to follow-up or termination of the observation).

All data are presented as the mean ± standard deviation. Student’s *t-*test was used for comparison of parameters between groups with GraphPad Prism 5 software (GraphPad Software, San Diego, CA, USA). Three levels of significance between groups (*p < 0.05; **p < 0.01; ***p < 0.001) were used, and differences for which *p* < 0.05 were considered to be significant.

## Results

### Identification and validation of TNFAIP1 as a downstream protein of the neddylation pathway

**Fig. 1:**
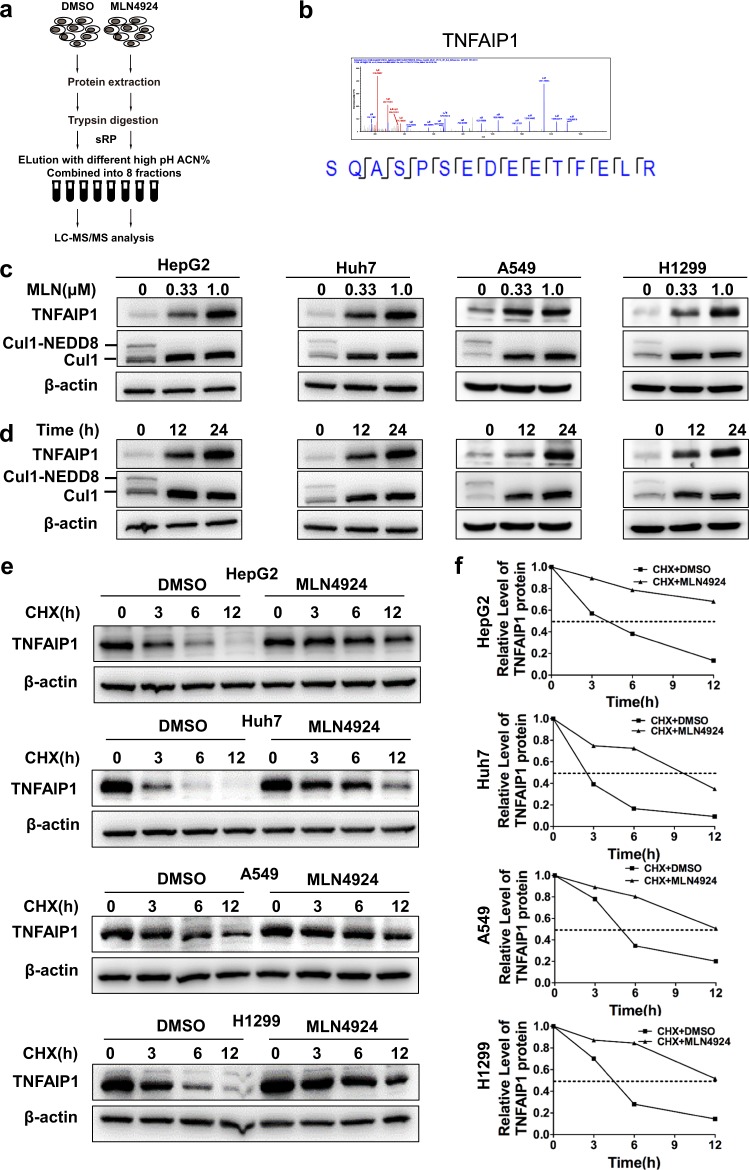
Identification and validation of TNFAIP1 as a novel downstream target of the neddylation pathway. **a** A schematic view of the label-free quantitative proteomics strategy based on mass spectra used in this study. The expression of up- and downregulated proteins is reported as the ratio of the peak intensity. **b** Representative tandem MS spectrum of the SQASPSEDEETFELR peptide showing that the cysteine was carbamidomethylated and the amino groups at the N-terminus and in the lysine were modified, as determined by label-free quantitative proteomics. **c**, **d** TNFAIP1 was accumulated upon MLN4924 treatment in a dose- and time-dependent manner. The Huh7, HepG2, A549, and H1299 human cancer cell lines were treated with MLN4924 at different doses for the indicated duration, harvested and subjected to western blot analysis using antibody against TNFAIP1. β-actin was used as the loading control. **e**, **f** The inhibition of neddylation with MLN4924 significantly extended the half-life of TNFAIP1. HepG2, Huh7, A549, and H1299 cells were pretreated with 1 µM MLN4924 for 12 h, after which the medium was exchanged with fresh DMEM containing 50 μg/ml cycloheximide (CHX) and 1 μM MLN4924. The cells were incubated for the indicated number of hours, and the cell lysates were subjected to western blot analysis with anti-TNFAIP1 Ab

A label-free quantitative proteomic strategy was carried out to identify up- and downregulated proteins upon neddylation inhibition with MLN4924 (a small-molecule inhibitor of the neddylation pathway) in HepG2 cells (Fig. 1a). A total of 8539 proteins were detected, among which 384 proteins were upregulated more than two-fold, including TNFAIP1, the expression of which was increased by 10.2-fold upon MLN4924 treatment (Fig. 1b). To further verify the proteomic results, we determined the protein level of TNFAIP1 by western blot analysis and found that TNFAIP1 expression was markedly increased in a dose- and time-dependent manner upon treatment with MLN4924^15^ in multiple cancer cell lines (Fig. 1c, d). Taken together, these findings demonstrate TNFAIP1 as a potential downstream target of the neddylation pathway.

To clarify the molecular mechanism of TNFAIP1 accumulation after blockade of the neddylation-CRL pathway, we first determined the TNFAIP1 turnover rate upon MLN4924 treatment. To this end, CHX was applied to block protein translation, and as shown in Fig. 1e, f, MLN4924 significantly delayed TNFAIP1 turnover and extended the half-life of TNFAIP1 in four cancer cell lines. These findings indicate that the neddylation-CRL pathway likely regulates TNFAIP1 degradation through the ubiquitin-proteasome system in cancer cells.

### CRL3 mediates the degradation and ubiquitination of TNFAIP1

**Fig. 2:**
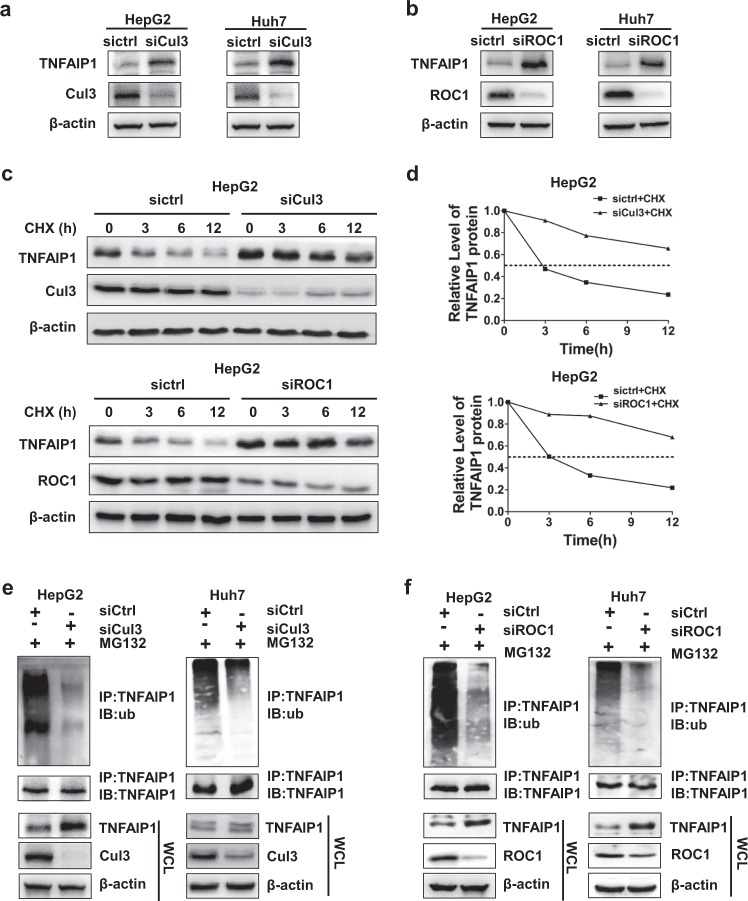
CRL3 mediates TNFAIP1 ubiquitination and degradation. **a**, **b** The Cul3-ROC1 complex regulateed the expression of TNFAIP1. **a** HepG2 or Huh7 cells were transfected with siRNA against *Cul3* for 96 h, lysed by protein lysis buffer and subjected to western blot analysis with anti-TNFAIP1 Ab. **b** HepG2 or Huh7 cells were transfected with siRNA against *ROC1* for 96 h, harvested and subjected to western blot analysis using antibody against TNFAIP1. **c**, **d** Cul3-ROC1 complex extended the half-life of TNFAIP1. **c**, HepG2 cells were transfected with siRNA against *Cul3* or *ROC1* for 84 h, after which the medium was exchanged with fresh medium containing 50 μg/ml CHX. The cells were incubated for the indicated number of hours, and the cell lysates were subjected to western blot analysis with antibody against TNFAIP1. **d** The expression of TNFAIP1 was quantified by densitometric analysis with ImageJ software. **e**, **f** Downregulation of *Cul3* or *ROC1* inhibited TNFAIP1 polyubiquitination in HepG2 and Huh7 cells. Cells were transfected with control siRNA or siRNA against *Cul3* or *ROC1* for 96 h, followed by treatment with MG132 for 2 h. The cells were extracted and subjected to immunoprecipitation with anti-TNFAIP1 Ab and western blot analysis using antibody against ubiquitin

Different combinations of 8 Cullin proteins and 2 ROC proteins constitute multiple CRL ubiquitin ligase subfamilies. To determine the exact CRL E3 ligase that regulates the ubiquitination and degradation of TNFAIP1 in cells, we first downregulated Cullin proteins with siRNAs. As shown in Fig. 2a, TNFAIP1 was accumulated only when *Cul3* was knocked down, and there was no obvious change in TNFAIP1 expression when the other *Cullin* family members were silenced under these experimental conditions (Supplementary Fig. 1a). TNFAIP1 was also accumulated in A549 and H1299 cell lines (shown in Supplementary Fig. 1b). As an indispensable subunit of CRL, ROC1 interacts with Cul3 to compose CRL3 complex. When ROC1 was knocked down, the activity of CRL was halted,^34^ subsequently inducing the accumulation of TNFAIP1 (Fig. 2b and Supplementary Fig. 1c).

After determining that CRL3 E3 ligase ablation induced TNFAIP1 accumulation, the turnover rate of TNFAIP1 upon CRL3 inactivation was investigated. Notably, we found that when *Cul3* or *ROC1* was knocked down with siRNA, TNFAIP1 turnover was significantly delayed (Fig. 2c), and the TNFAIP1 protein half-life was extended (Fig. 2d).

Next, we detected the level of TNFAIP1 ubiquitination upon downregulation of *Cul3* or *ROC1*. As shown in Fig. 2e, silencing *Cul3* significantly impaired TNFAIP1 polyubiquitination in both HepG2 and Huh7 cells. Consistently, downregulation of *ROC1* also significantly inhibited the polyubiquitination of TNFAIP1 in both HepG2 and Huh7 cells (Fig. 2f). Taken together, these findings indicate that Cul3-ROC1 ubiquitin ligase (CRL3) targets TNFAIP1 for ubiquitination and degradation in cancer cells.

### BTBD9, an adaptor of CRL3, regulates the degradation of TNFAIP1

**Fig. 3:**
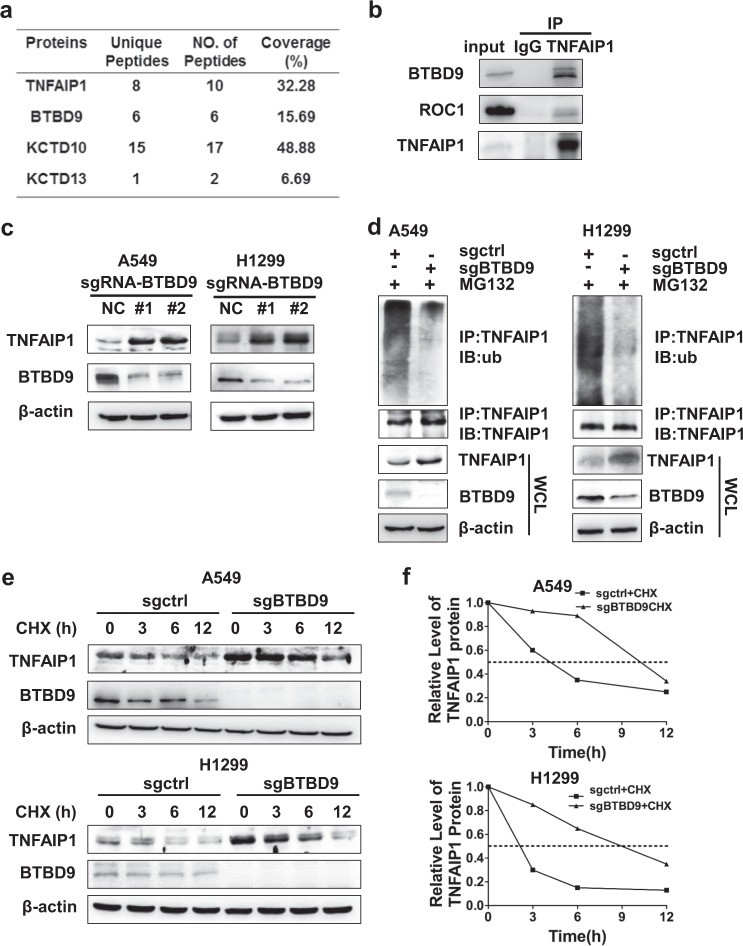
BTBD9, an adaptor of CRL3, mediates the degradation of TNFAIP1. **a** Endogenous immunoprecipitation with anti-TNFAIP1 antibody and subsequent mass spectrometry were used to identify the adaptor of CRL3, which contains the BTB domain and regulates TNFAIP1 degradation. **b** Immunoprecipitation and western blot analysis were used to verify the interaction between TNFAIP1 and BTBD9. **c**, **d** A CRISPR/Cas9 system was established to downregulate the expression of *BTBD9*, and the expression and polyubiquitination of TNFAIP1 was determined in A549 and H1299 cells. **c** Cell lysates were collected and subjected to western blot analysis with antibody against TNFAIP1. **d** Cell lysates were harvested and subjected to immunoprecipitation with anti-TNFAIP1 Ab and western blot analysis using antibody against ubiquitin. **e** A549 or H1299 cells were treated with CHX (50 μg/ml) for the indicated number of hours, and cell lysates were subjected to western blot analysis with antibody against TNFAIP1. **f** The relative expression of TNFAIP1 was quantified by densitometric analysis with ImageJ software

Adaptor proteins of CRL3 directly interact with certain substrates and determine the specific of CRL3 for those substrates. To identify the specific adaptor of CRL3 that mediates the degradation of TNFAIP1, endogenous immunoprecipitation with TNFAIP1-specific Ab and mass spectra were performed to identify the BTB domain-containing proteins that directly interact with TNFAIP1. In addition to TNFAIP1, three BTB domain-containing proteins, BTBD9, KCTD10, and KCTD13, were identified through this screen (Fig. 3a). As shown in Fig. 3b, BTBD9 interacted with TNFAIP1. Next, the CRISPR/Cas9 system was applied to downregulate the expression of *BTBD9*, which subsequently induced the accumulation of TNFAIP1 (Fig. 3c). Moreover, as shown in Fig. 3d, polyubiquitination of TNFAIP1 was significantly impaired upon downregulation of *BTBD9* in both A549 and H1299 cells. Consistently, the half-life of TNFAIP1 was also extended when *BTBD9* was silenced with the CRISPR/Cas9 system (Fig. 3e, f). However, no obvious change in TNFAIP1 was observed when *KCTD10* or *KCTD13* was silenced under these experimental conditions, thereby refuting the potential role of KCTD10 or KCTD13 in regulating TNFAIP1 (Supplementary Fig. 2). Together, these findings indicate that CRL3^BTBD9^ targets TNFAIP1 for ubiquitination and degradation in cancer cells.

### BTBD9 suppresses cancer cell migration by promoting TNFAIP1 degradation

**Fig. 4:**
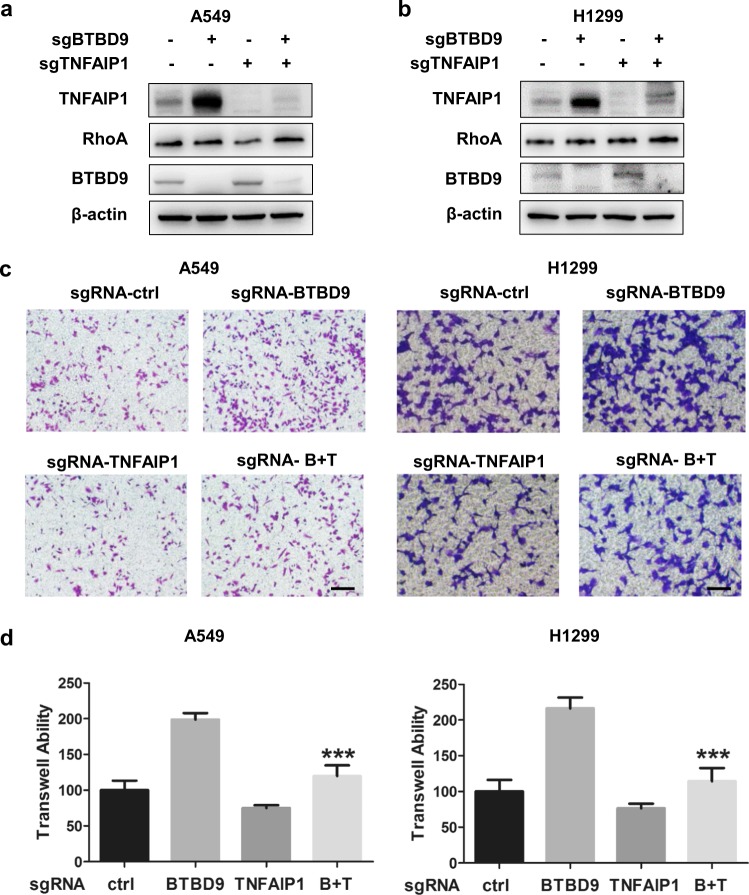
Reduced expression of BTBD9 leads to TNFAIP1 accumulation, promoting metastasis in lung cancer. **a** The effects of BTBD9 and TNFAIP1 knockdown in A549 cells were confirmed using western blotting. **b** The effects of BTBD9 and TNFAIP1 knockdown in H1299 cells were confirmed using western blotting. **c**, **d** The Transwell assay was used to detect the migration ability of A549 and H1299 cells in which *TNFAIP1* and *BTBD9* were downregulated individually or together (scale bar = 100 μm). Data are presented as the mean ± SD (*n* = 5), *p* < 0.001 vs the sgRNA-BTBD9 group. These data are representative of three independent experiments, the results of which showed similar trends

By the results mentioned above, BTBD9 was identified for the first time as an adaptor that regulates substrate degradation. However, the physiological function of BTBD9 in tumorigenesis and development is not yet fully understood. To further elucidate the function of BTBD9 and TNFAIP1 in lung cancer, the CRISPR/Cas9 system was used to knockdown *BTBD9* or *TNFAIP1* individually and simultaneously (Fig. 4a, b). As shown in Fig. 4c, d, a Transwell assay demonstrated that the migration of lung cancer cells was elevated when *BTBD9* was silenced. *TNFAIP1* knockdown partially rescued the increased cell migration induced by *BTBD9* downregulation in both A549 and H1299 cells. Altogether, our results suggest that BTBD9 suppresses cancer cell migration by promoting TNFAIP1 degradation.

### TNFAIP1 is overexpressed in lung cancer and predictes poor overall survival of lung cancer patients

**Fig. 5:**
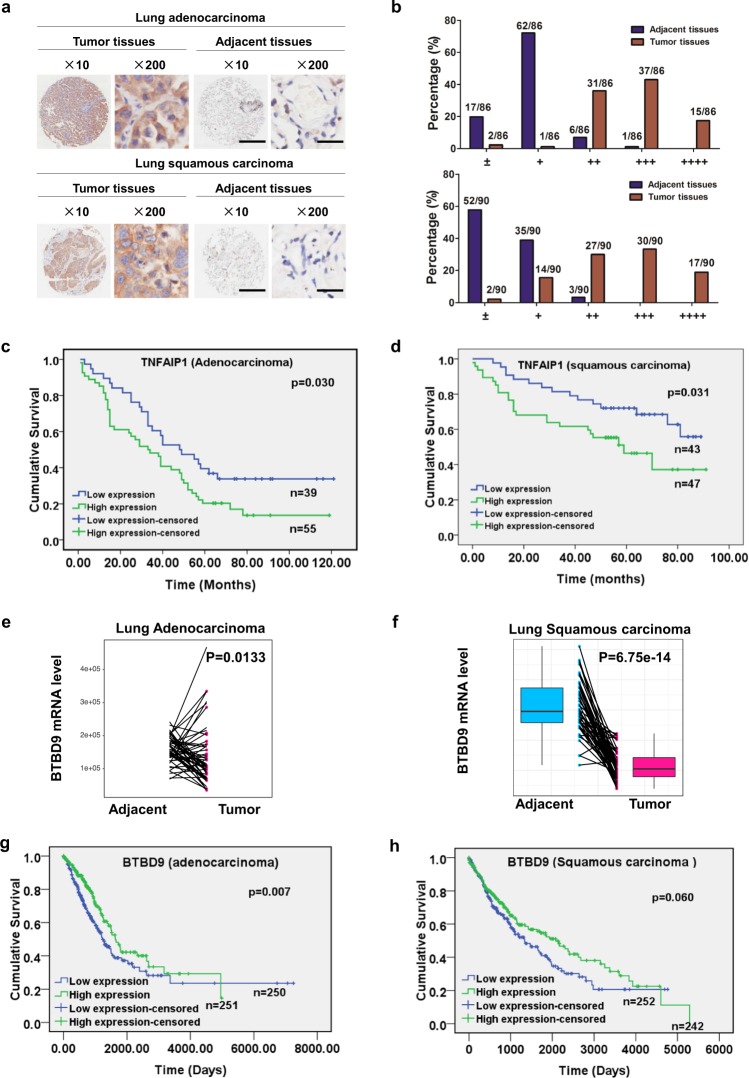
BTBD9 and TNFAIP1 are negatively correlated in lung cancer tissues. **a** Immunohistochemical staining of human lung adenocarcinoma and squamous carcinoma tissue arrays using specific antibody against TNFAIP1 (scale bar for the images at ×10, 500 μm; scale bar for the images at ×200, 25 μm). Samples were scored according to the percentage of positively stained tumor cells and the staining intensity for TNFAIP1. **b** The samples were classified into five groups based on staining intensity from the weakest (±, group 1) to the strongest (++++, group 5) staining intensity. **c**, **d** Kaplan–Meier curves showing the overall survival rate of patients with lung adenocarcinoma and squamous carcinoma according to the expression level of TNFAIP1 (log-rank test) are shown. **e**, **f** mRNA levels of *BTBD9* in lung adenocarcinoma and lung squamous carcinoma were analyzed according to data from TCGA database. **g**, **h** Kaplan–Meier curves for the overall survival rate of patients with lung adenocarcinoma and squamous carcinoma according to the *BTBD9* mRNA level (log-rank test) are shown

To estimate the status of TNFAIP1 in human lung cancer, IHC staining of human lung adenocarcinoma and squamous carcinoma tissue arrays containing primary tumor tissues and the paired adjacent normal tissues was first performed to determine the expression levels of TNFAIP1. Based on the staining intensity, we classified the samples into five groups with increasing staining intensity from the weakest to the strongest. Classification analysis demonstrated that the expression of TNFAIP1 was lower (falling into group 1 and group 2) in the majority of adjacent normal tissues, whereas it was higher (falling into group 3 to group 5) in the majority of lung tumor tissues (Fig. 5a, b). TNFAIP1 was overexpressed in both lung adenocarcinoma (Fig. 5a, b, upper) and squamous cell carcinoma tissues (Fig. 5a, b, lower) compared with adjacent normal tissues.

In addition, Kaplan–Meier analysis showed that the overall survival rate was lower in lung adenocarcinoma and squamous cell carcinoma patients with high TNFAIP1 expression than in patients with low TNFAIP1 expression (in lung adenocarcinoma: *p* = 0.030; in lung squamous cell carcinoma, *p* = 0.031, log-rank test) (Fig. 5c, d). High expression of TNFAIP1 was associated with poorer overall survival of lung adenocarcinoma and squamous cell carcinoma patients, as shown by univariable survival analyses (TNFAIP1 in lung adenocarcinoma: HR = 1.703, 95% CI = 1.042 to 2.782, *p* = 0.0034; TNFAIP1 in lung squamous carcinoma: HR = 0.494, 95% CI = 0.257 to 0.952, *p* = 0.035). Taken together, these findings demonstrate that TNFAIP1 is overexpressed in human lung cancer and associated with reduced overall survival of patients.

### The mRNA level of BTBD9 is downregulated, which predictes poor overall survival in lung cancer patients

To evaluate the expression level of BTBD9 in lung cancer, data from TCGA database were downloaded, and bioinformatics analysis demonstrated that the mRNA level of *BTBD9* was downregulated in human lung adenocarcinoma and lung squamous carcinoma (Fig. 5e, f). Meanwhile, prognostic analysis showed that the low mRNA expression of *BTBD9* was positively correlated with poor prognosis in lung cancer patients (in lung adenocarcinoma: *p* = 0.007; in lung squamous cell carcinoma, *p* = 0.060, log-rank test) (Fig. 5g, h). These findings suggest that BTBD9 is downregulated and associates with reduced overall survival of patients.

## Discussion

TNFAIP1 regulates a variety of physiological and pathological processes, including tumorigenesis and cancer cell migration.^24,26–28,35,36^ However, its regulatory mechanism has not yet been fully elucidated. In the present study, we demonstrated that the ROC1-Cul3-BTBD9 complex is a novel E3 ligase that modulates the polyubiquitination and turnover of TNFAIP1. Functionally, we found that BTBD9 suppressed lung cancer cell migration by promoting TNFAIP1 degradation in vitro. Moreover, low expression of BTBD9 was associated with reduced overall survival of patients, indicating the tumor-suppressive role of BTBD9. In contrast, TNFAIP1 was overexpressed in lung cancer, which predicted poor prognosis in lung cancer patients. These findings indicate that dysregulation of CRL3^BTBD9^ may halt TNFAIP1 degradation in lung cancer to regulate the migration and metastasis of lung cancer.

BTBD9, a BTB domain-containing protein, is significantly associated with susceptibility to sleep disturbance and restless leg syndrome (RLS).^37–40^ However, there has been no study about its role in cancer or its role as an adaptor of CRL3 E3 ligases that modulates the degradation of tumor suppressors or oncoproteins. In this study, BTBD9 was first identified as an adaptor of CRL3 that regulates the ubiquitination and degradation of its substrate protein TNFAIP1, as demonstrated by the following: (1) TNFAIP1 and BTBD9 interacted, (2) downregulation of *BTBD9* delayed the turnover of TNFAIP1, and (3) *BTBD9* knockdown diminished the polyubiquitination of TNFAIP1. We hypothesize that BTBD9, an adaptor of CRL3, could regulate the polyubiquitination and degradation of other substrates, but further studies are needed to identify these substrates.

However, the silencing of neither *BTBD9* nor *TNFAIP1* affected cancer cell proliferation, and our study systematically demonstrated that the downregulation of BTBD9 halted TNFAIP1 degradation in lung cancer and subsequently drove lung cancer cell metastasis. To elucidate the molecular mechanism by which BTBD9 regulates TNFAIP1 to affect lung cancer cell migration, we detected the expression level of RhoA, which was reported to be regulated by TNFAIP1 and responsible for stress fiber formation. Unfortunately, in this study, we found that *BTBD9* knockdown had no effect on the expression of RhoA. Therefore, we speculated that BTBD9 regulated the degradation of TNFAIP1 and affected the metastasis of cancer cells in a RhoA-independent manner. As expected, GO biological process analysis of TNFAIP1-interacting proteins revealed that TNFAIP1 was associated with the cytoskeleton. Among the proteins enriched in these pathways, 6 proteins were involved in cytoskeleton regulation, as shown by analysis of a protein-protein interaction network including FLNB, TLN1, RACGAP1, KIF23, SNX9, and MYH10^41–47^. We speculated that BTBD9 affected the expression of these proteins by modulating TNFAIP1 degradation and altering the metastatic ability of lung cancer cells. The precise regulatory mechanism needs further confirmation.

**Fig. 6:**
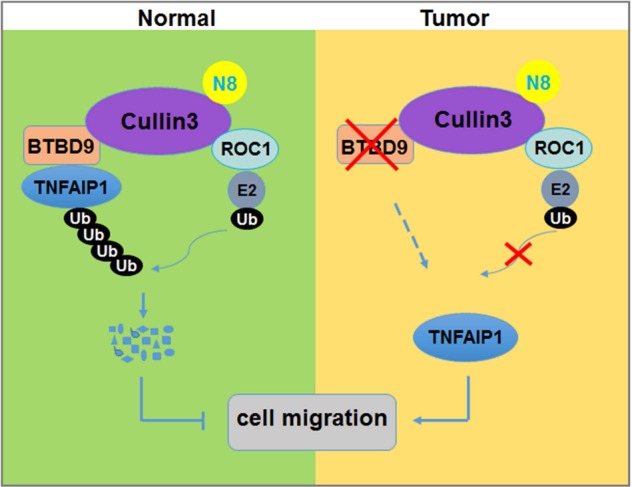
Working model. Cul3-ROC1-BTBD9 ubiquitin ligase complex regulates the polyubiquitination and degradation of TNFAIP1 in normal cells, whereas in a cancer environment, low BTBD9 expression leads to dysregulated TNFAIP1 degradation and enhanced cancer cell migration

In summary, our study identified TNFAIP1 as a novel substrate of CRL3^BTBD9^. Next, CRL3^BTBD9^ E3 ubiquitin ligase was determined to suppress cancer cell migration by mediating TNFAIP1 degradation. Finally, we clarified that TNFAIP1 is overexpressed in the development of lung cancer due to dysfunction of CRL3 adaptor protein BTBD9, which is also associated with poor prognosis in lung cancer patients (Fig. 6). Altogether, our study highlights the pivotal role of the CRL3-BTBD9-TNFAIP1 axis in lung carcinogenesis and development.

## Supplementary information

TNFAIP1 supplementary materials
