# Supplementary material for: The CRL3BTBD9 E3 ubiquitin ligase complex targets TNFAIP1 for degradation to suppress cancer cell migration
Source: Signal Transduct Target Ther. 2020 Apr 24;5:42. doi: 10.1038/s41392-020-0140-z (PMC7181851; doi:10.1038/s41392-020-0140-z)
Supplement: Supplementary file 1 — TNFAIP1 supplementary materials [file 41392_2020_140_MOESM1_ESM.docx]

Supplementary Materials for

**CRL3^BTBD9^ E3 ubiquitin ligase complex targets TNFAIP1 for degradation to suppress cancer cell migration**

Lihui Li^1#*^, Wenjuan Zhang^1, 2#^, Yue Liu^3^, Xiaojun Liu^2^, Lili Cai^1^, Jihui Kang^1^, Yunjing Zhang^1^, Wenlian Chen^1^, Changsheng Dong^1^, Yanmei Zhang^3^, Mingsong Wang^4^, Wenyi Wei^5^, Lijun Jia^1*^

**Corresponding author:** Lijun Jia, Email: [ljjia@shutcm.edu.cn](mailto:ljjia@shutcm.edu.cn); Lihui Li, Email: [dm-li@163.com](mailto:dm-li@163.com)

**This PDF file includes:**

Materials and Methods

Figures. S1 to S3

Supplementary Figure Legends

**Materials and Methods**

**Cell lines and reagents**

Human hepatocellular carcinoma cell lines Huh7 and HepG2, lung cancer cell lines A549 and H1299 were obtained from the American Type Culture Collection, and were cultured in DMEM or RPMI-1640 medium (Thermo Fisher Scientific), supplemented with 10% FBS and 1% penicillin- streptomycin, at 37 °C with 5% CO_2_. MLN4924 was synthesized and used as described previously. MG132 (sigma) and cycloheximide (CHX, Cell Signaling Technology) were kept at -20 °C.

**RNA interference**

Huh7 or HepG2 cells were transfected with siRNA oligonucleotides using Lipofectamine 2000 reagent (Life Technologies, Invitrogen, CA) according to the manufacturer’s instruction. The sequences of siRNAs are as follows: for *ROC1*, 5’-GACUUUCCCUGCUGUUACCUAATT-3’; for *Cul3*, 5’- TTGACGTGAACTGACATCCACATTC-3’; for *Cul1*, 5’- CUAGAUACAAGAUUAUACAUGCGG-3’; for *Cul2*, 5’ -GGGCUUCUACAAGAGUGGGAAGUTT-3’; for *Cul3*, 5- TTGACGTGAACTGACATCCACATTC-3; for *Cul4a*, 5’ -GAAGAUUAACACGUGCUGGdTdT-3’; for *Cul4b*, 5’-GGUGAACACUUAACAGCAAdTdT-3’; for *Cul5*, 5’-CAGCTGGTTATTGGAGTAAGA-3’; for *KCTD10*, 5’-CCAGCAAUUCUGACGACAAUTT-3’; for *KCTD13*, 5’-UUAAGUAGGUUGUCAUCUGAAGUGC**-**3’; and for control scrambled siRNA 5’-UUCUCCGAACGUGUCACGUTT-3’. All of the above siRNAs were ordered from GenePharma (Shanghai, China).

**Generation of stable cell lines by CRISPR/Cas9 system**

For packaging lenti-virus used in TNFAIP1 and *BTBD9* knockdown, two guide RNA sequences specifically against *TNFAIP1* and *BTBD9* were inserted into vector lenti-guide-puro, respectively. 293T cells were co-transfected with lenti-viral vectors lenti-guide-puro and packaging vectors AGP091 and AGP090 as described previously ^1^. Forty-eight hours after transfection, the viral supernatants were collected, filtered, and infected lung cancer cell lines as described previously ^1^. Six hours after incubation, the viral supernatant was replaced with normal DMEM with 10% FBS.

**Transwell migration assays**

The standard transwell migration assay, using a transwell polycarbonate filter, was performed to analyze the cell migration abilities.according to our published methods ^1,2^. Briefly, cells were suspended with fetal bovine serum (FBS)-free DMEM and diluted to 4×10^5^ cells/mL. 100 μL cell suspension was added into the upper chambers. 600 μL DMEM, containing 20% FBS was added into the lower chambers. After 24 hours incubation, the upper chambers were fixed in 4% formalin for 15 minutes and then stained with 0.1% crystal violet for 30 minutes. Cells that passed through the polycarbonate membrane of the well were counted under a Leica microscope. The representative results of three independent experiments with similar trends are presented.

**Collection of Lung Cancer Tissues and Clinicopathological Characteristics of Patients**

All patients underwent surgery, followed by treatment in accordance with the National Comprehensive Cancer Network clinical practice guidelines. Fresh primary lung cancer tissues and adjacent lung tissues were collected from lung adenocarcinoma and lung squamous cell carcinoma patients undergoing resection from July 2004 to May 2008 at the Taizhou Hospital (Taizhou, China). Histological diagnosis and tumor–node–metastasis staging of cancer were determined in accordance with the American Joint Committee on Cancer manual criteria for lung cancer. Written informed consent regarding tissue and data use for scientific purposes was obtained from all participating patients. The study was approved by the Research Ethics Committee of Taizhou Hospital.

**Real-Time Polymerase Chain Reaction Analyses**

Total RNA was isolated using the Trizol reagent (Invitrogen, Carlsbad, CA) according to the manufacturer’s instructions and treated with RNase-free DNase. The reverse transcription reaction was performed on 2.5 μg of total RNA per sample using the PrimerScript reverse transcription reagent kit (TaKaRa, Shiga, Japan) according to the manufacturer’s protocol. After reverse transcription, the real-time polymerase chain reaction (PCR) was performed using the Power SYBR Green PCR MasterMix (Applied Biosystems, Foster City, CA) on the ABI 7500 thermocycler (Applied Biosystems) following the instrument manual.

Figure. S1.


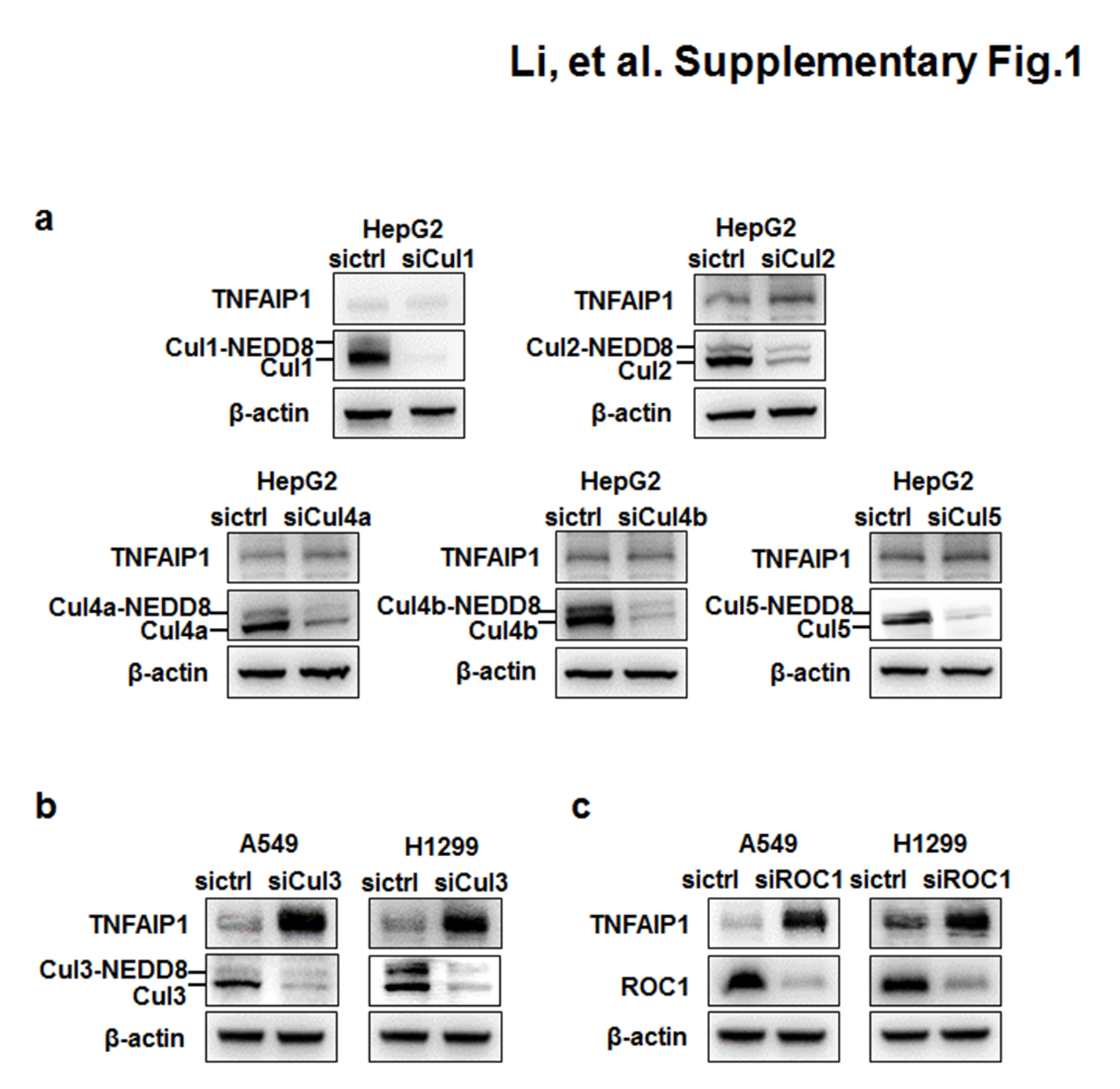


Figure. S2.


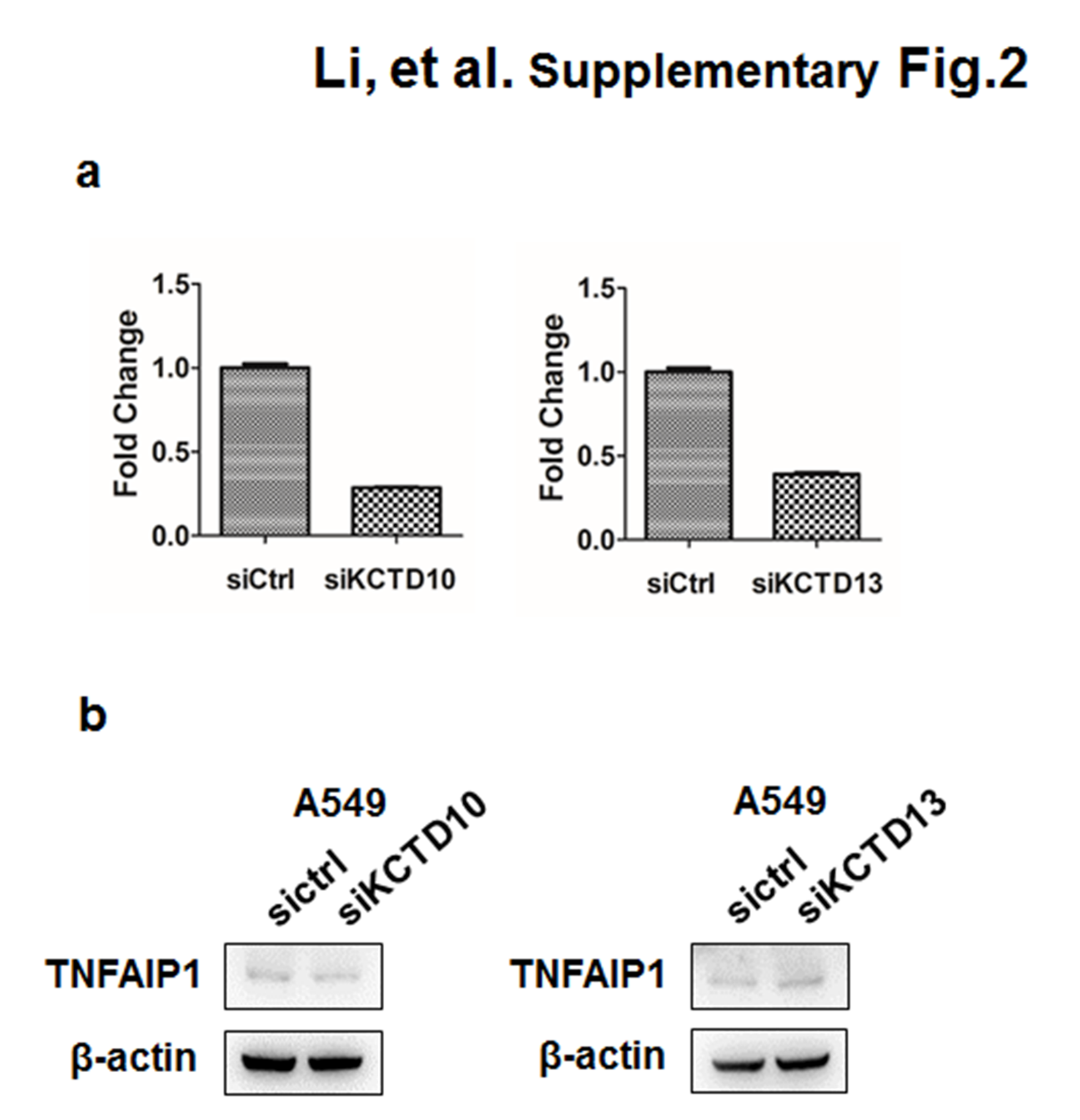


Figure. S3.


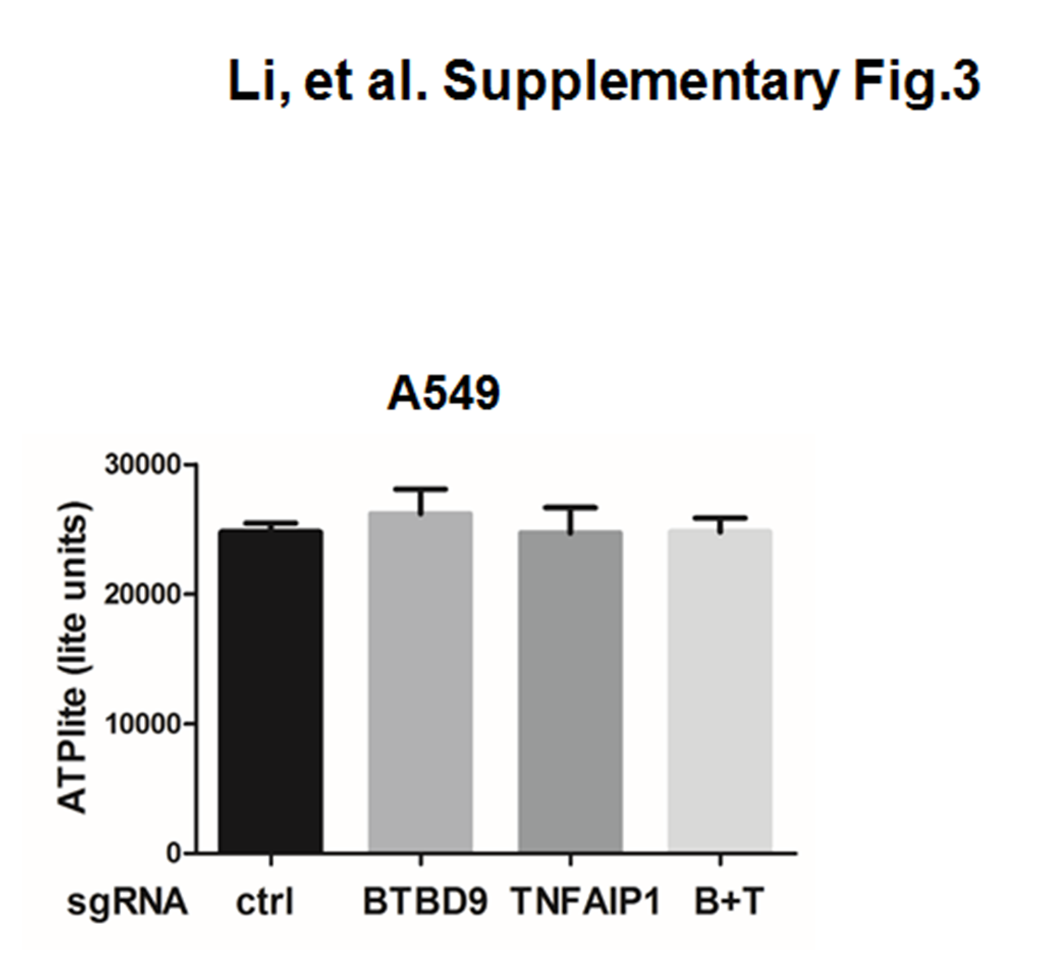


Supplementary Figure Legends

**Supplementary Figure 1. Cullin3-ROC1 E3 ligase (CRL3) governs TNFAIP1 expression.**

a, HepG2 cells were transfected with siRNAs against *Cul1, Cul2, Cul4a, Cul4b, Cul5* for 96 hours, respectively. Afterwards, cell lysates were harvested and subjected to western blot analysis using antibody against TNFAIP1. β-actin was used as the loading control. b and c, A549 and H1299 cells were transfected with siRNAs against *Cul3* (b) and *ROC1* (c) for 96 hours, respectively. Cell lysates were harvested and subjected to western blot analysis using antibody against TNFAIP1.

**Supplementary Figure 2. KCTD10 and KCTD13, two BTB domain containing proteins, had no effect on TNFAIP1 expression.** A549 cells were transfected with siRNAs against *KCTD10* or *KCTD13*, respectively. a, 24 hours later, total RNA was harvested and subjected to the real time PCR analysis. b, 96 hours later, cell lysates were harvested and subjected to western blot analysis using antibody against TNFAIP1. β-actin was used as the loading control.

**Supplementary Figure 3. BTBD9 and TNFAIP1 had minimal effect on A549 cell proliferation.** A549 cells were infected lenti-virus against *BTBD9* or *TNFAIP1*. 1500 cells were seeded in 96-well plates in triplicates for 72 hours and then lysed for ATPlite assay.

1 Li, L. *et al.* Validation of NEDD8-conjugating enzyme UBC12 as a new therapeutic target in lung cancer. *EBioMedicine*. **45**, 81-91, (2019).

2 Li, L. *et al.* Overactivated neddylation pathway as a therapeutic target in lung cancer. *J Natl Cancer Inst*. **106**, dju083, (2014).
